# Pesticide poisoning in non-fatal deliberate self-harm: A public health issue: Study from Sundarban delta, India

**DOI:** 10.4103/0019-5545.37666

**Published:** 2007

**Authors:** A. N. Chowdhury, Sohini Banerjee, Arabinda Brahma, M. K. Biswas

**Affiliations:** Institute of Psychiatry, 7 D. L. Khan Road, Kolkata - 700 025, India

**Keywords:** Community psychiatry, deliberate self-harm, pesticide poisoning, primary care, Sundarban

## Abstract

**Background:**

Non-fatal deliberate self-harm (DSH), particularly with pesticides, is a major public health problem in many developing countries of the world. Agriculture is the primary occupation of most people living in the Sundarban region in West Bengal, India. Pesticides are extensively used in agriculture, and these agents are most frequently used in DSH.

**Aim:**

This study aimed to identify the nature of methods and agents used in non-fatal DSH attempts in the Sundarban area under South 24 Parganas district of West Bengal.

**Materials and Methods:**

Detailed demographic and clinical data on DSH cases of 13 Block Primary Health Centres' (BPHCs') admission registers were analyzed. One Focus Group Discussion (FGD) with the *Panchayat Samiti* of each block (totally 13 FDGs) was conducted to elicit the *Samiti* members' perception about the problem of pesticide-related DSH or suicide in the region.

**Results:**

A total of 5,178 (1,887 male and 3,291 female) subjects were admitted at the BPHCs during the study period from 1999 to 2001. Organophosphorous pesticide poisoning was found to be the most common method (85.1%) in DSH. This emphasizes the importance of developing an urgent poisoning-prevention program with a special focus on improving clinical services, as well as initiating farmers' education programs focusing on safe pesticide practices at the primary-care level.

## INTRODUCTION

Deliberate self-harm, particularly with pesticides, is a major public health problem, especially in developing countries.[1–3] Some studies from India have reported similar findings.[4–7] However, documentation on this issue is often not available from very remote areas such as the Sundarban region in West Bengal, India.

Sundarban is the largest delta in the world and famous for its mangrove vegetation and Royal Bengal tigers. Indian Sundarban is the southernmost part of the state of West Bengal, at the confluence of the river Hooghly and the Bay of Bengal. Sundarban region under South 24 Parganas district is having 13 community-development blocks (CDBs). Each CDB has an average population of 1–1.5 lakh and a nodal health service facility known as Block Primary Health Centre (BPHC). Sundarban is a backward region by all yardsticks of socioeconomic development. The literacy rate and per capita income is much lower than the state average, and about 88.5% inhabitants are dependent on agriculture. Life in the entire region is subject to various ecological hazards. Coastal erosion, tidal ingress, sand encroachment and salinity of soil are the few factors that adversely affect agriculture.[8]

**Figure 1:**
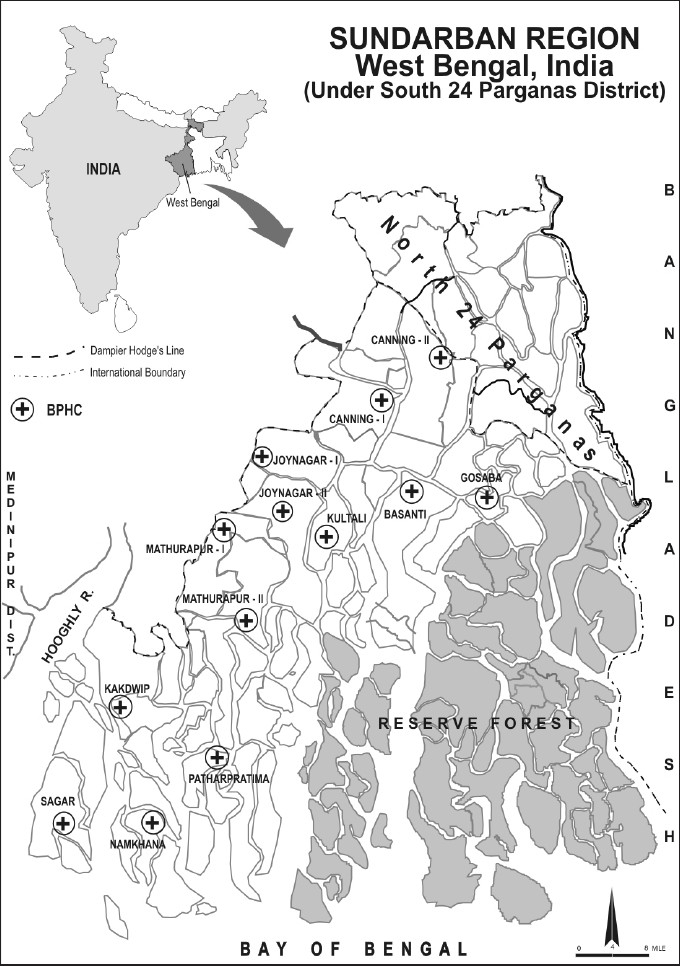
Sundarban region, West Bengal

In course of a previous mental-health research in the Sundarban region, the local community expressed worry about DSH and suicide, particularly with pesticides. To study the extent of DSH in the region, research was carried out in all the BPHCs of the 13 CDBs, viz., Basanti, Canning I and Canning II, Gosaba, Joynagar I, Joynagar II, Kakdwip, Kultali, Mathurapur I, Mathurapur II, Namkhana, Patharpratima and Sagar [Figure 1].

## MATERIALS AND METHODS

### DSH data

The research team made 3-4 visits to each of the 13 BPHCs between January 2002 and April 2002. Information on DSH was collected for the period from 1^st^ January 1999 to 31^st^ December 2001 from admission and emergency registers. Data on suicides and accidental poisoning were also collected, which have been reported elsewhere. In this article, the different types of agents used in DSH are discussed.

### Participatory research observation

One Focus Group Discussion (FGD) with the *Panchayat Samiti* of each block (totally 13 FDGs) was conducted to elicit the *Samiti* members' perception about the problem of pesticide-related DSH or suicide in the region. The opinion of the *Samiti* members about sociocultural dynamics of DSH and pesticide use was collected. *Panchayat Samiti* is the locally elected democratic body which acts as the local administrative organ in the three-tire *Panchayat* system, responsible for implementation of all developmental work for the blocks, including health services.

## RESULTS

There were 1,691 cases (male 579, female 1,112) of DSH in 1999, 1,775 cases (male 668, female 1107) of DSH in 2000 and 1,712 cases (male 640, female 1072) of DSH in 2001. Table 1 shows the sex-wise distribution of all the combined cases (5,178 cases: 1,887 male, 3,291 female) according to the BPHCs. The maximum number of cases were admitted in Joynagar I (1,286), followed by Canning I (898) BPHC. Females (63.6%) had clear predominance over males (36.4%) in all the three years.

**Table 1:** BPHC-wise distribution of methods in non-fatal DSH cases (1999-2001)

Blocks/DSH number	Male							Female							
		
	Hang	Burn	Drown	HCh	IP	OP	Oth	Hang	Burn	Drown	Inj	HCh	IP	OP	Oth
Basanti	1	1				7		1	1				1	12	
n 24 (M 9/F 15)	11.1	11.1				77.8		6.7				6.7	6.7	80.0	
Canning I	7	12	1	5	6	272	18	3	25	1		10	18	502	18
n 898 (M 321/F 577)	2.2	3.7	0.3	1.6	1.9	84.7	5.6	0.5	4.3	0.2		1.7	3.1	87.0	3.1
Canning II		1				10			1			1	2	18	
n=33 (M 11/F 22)		9.1				90.9			4.5			4.5	9.1	81.8	
Gosaba		2				29			4			2	1	44	
n=82 (M 31/F 51)		6.5				93.5			7.8			3.9	2.0	86.3	
Jaynagar I		2		4	1	446	4	1	2			10	4	803	9
n=1286 (M 457/F 829)		0.4		0.9	0.2	97.6	0.9	0.1	0.2			1.2	0.5	96.9	1.1
Jaynagar II	3			3		84	28	1	1	1		6	3	140	40
n=310 (M 118/F 192)	2.5			2.5		71.2	23.7	0.5	0.5	0.5		3.1	1.6	72.9	20.8
Kakdweep	7	5	2	4	5	166	9	4	10	1		19	16	267	8
n=523 (M 198/F 325)	3.5	2.5	1.0	2.0	2.5	83.8	4.5	1.2	3.1	0.3		5.8	4.9	82.2	2.5
Kultali						32	7						1	89	24
n=153 (M 39/F 114)						82.1	17.9						0.9	78.1	21.1
Mathurapur I	2	5	1	7	2	146	5	2	4			15	7	245	27
n=468 (M 168/F 300)	1.2	3.0	0.6	4.2	1.2	86.9	3.0	0.7	1.3			5.0	2.3	81.7	9.0
Mathurapur II	2	2	1	4		193	6	2	9	2	1	8	3	291	11
n=535 (M 208/F 327)	1.0	1.0	0.5	1.9		92.8	2.9	0.6	2.8	0.6	0.3	2.4	0.9	89.0	3.4
Namkhana	1	1			2	80	24	3	1			3	30	114	23
n=282 (M 108/F 174)	0.9	0.9			1.9	74.1	22.2	1.7	0.6			1.7	17.2	65.5	13.2
Patharpratima		1		4	1	71	22	1	1	1		5	8	114	36
n=265 (M 99/F 166)		1.0		4.0	1.0	71.7	22.2	0.6	0.6	0.6		3.0	4.8	68.7	21.7
Sagar				12	5	97	6	1				32	22	135	9
n=319 (M 120/F 199)				10.0	4.2	80.8	5.0	0.5				16.1	11.1	67.8	4.5
Total	23	32	5	43	22	1633	129	19	59	6	1	111	116	2774	205
n=5178 (M 1887/F 3291)	1.2	1.7	0.3	2.3	1.2	86.5	6.8	0.6	1.8	0.2	0.03	3.4	3.5	84.3	6.2

HCh = Household chemicals, IP = Indigenous poisons, OP = Organophosphorus, Inj = Injury (Self-inflicted), Oth = Others. Figures in the second row in each box represent percentage

Table 2 shows the nature of different methods/ agents used in DSH attempt. Poisoning, especially by organophosphorous pesticide, was the commonest (85.1%), with a slight male preponderance (86.5%) over females (84.3%). “Others” (unknown poisoning and medicines like sedatives, homeopathic medicine, etc.) comes next (6.5%), followed by household chemicals (3.0%) like kerosene, rat killer, lice killer, etc.; and indigenous poisons (2.7%) like oleander seeds, *dhatura* seeds, etc. Traditional methods like self-immolation (1.8%), hanging (0.8%) and drowning (0.2%) were attempted by very few subjects.

**Table 2:** Distribution of methods/agents in non-fatal DSH cases (1999-2001)

Method/Agents	Male		Female		Total	
			
	n 1887	%	n 3291	%	n 5178	%
Pesticide Poisoning	1633	86.5	2774	84.3	4407	85.1
Burning	32	1.7	59	1.8	91	1.8
Drowning	5	0.3	6	0.2	11	0.2
Household chemical	43	2.3	111	3.4	154	3.0
Hanging	23	1.2	19	0.6	42	0.8
Indigenous poisoning	22	1.2	116	3.5	138	2.7
Self injury		0.0	1	.03	1	.02
*Other	129	6.8	205	6.2	334	6.5

* prescribed medicine, alcohol, unknown poisoning.

Table 3 shows the summary findings from the focus group discussions with 13 *Panchayat Samities*, which mostly pinpoint the issue of DSH with pesticide poisoning as an emerging public health agenda in the region. They also endorsed the opinion that varied types of psycho-social stressors, especially among the young married females, are positively linked with suicidal behaviors. All *Panchayat Samities* felt the need for more empowerment of the *Panchayat* to supervise the local trade of pesticides, along with imposition of more strict regulation by the administration on pesticide sale.

**Table 3:** Summary findings of the FGDs with Panchayat Samities

Deliberate self-harm and suicide by pesticide poisoning is quite common in the block, and it becomes a major health problem in the entire Sundarban region. Though it is seen in both sexes, frequency is higher among females, especially among young married females. Torture of women is an important cause behind many deliberate self-harm attempts. Family conflict and economic loss may be an important reason among the males. Recently deliberate self-harm is noted among school-going boys and girls, and mostly they are related with failure in love or examination failure. Exposure of younger population to sex- and violence-related movies in video parlors is highly influential in mitigating the development of alcohol habit, indecent sexual behavior and torture and violence of women in a section of the population. Easy availability of pesticides is a dangerous situation prevailing in this entire region. Farmers' education on safe use and storage of pesticide is an important method to create public awareness. Panchayat should have some powers in the regulation of local pesticide market. There is a definite lack of appropriate caution or knowledge of safe storage of pesticides among the farmers. Opportunities for health service should be available at the community level. Timely family intervention may save many lives, if arranged. Governmental steps concerning pesticide licensing or legal proceedings in cases of dowry-related deliberate self-harm and suicide should be handled more strictly.

## DISCUSSION

In many Asian countries, hanging, self-immolation and jumping from height are common methods of self-harm.[9] However, in the present study, deliberate pesticide poisoning was found to be the most frequent method. Similar finding was reported earlier from Sagar island of Sundarban region.[10] This study and the concurrent participatory observations in this region revealed some interesting findings that need the attention of mental-health providers. These are briefed as follows:

### Availability of pesticides

Agriculture is the main livelihood of people. Sundarban is remarkably known for its saline soil and varieties of pests and insects. An inverse relation was observed between increase of population and decrease of per-capita cultivable land in successive years. To compensate for the low yield of crops, aggressive use of fertilizers, pesticides and insecticides is the general rule for the farmers in the region. This invites easy availability of different varieties of organophosphorous pesticides through an unregulated, open local market. Most of the farmers lack proper education about storage and use of these lethal chemicals; they keep pesticides carelessly in their home within the easy access of other family members, including children. There is also no control on sale or purchase of pesticides. Numerous unregistered pesticide shops are operating in the entire region. Pesticides are sold even from the grocery shops in the villages. To site an example, the small island of Sagar has 114 registered and over 210 unregistered shops.[10] The present study found that “unknown poisoning” is responsible for a good number of DSH cases. These unregistered shops sell various kinds of pesticides that are recognized only by local brand names, not by the chemical compositions. It poses a practical difficulty for the physicians at the BPHC – to identify the poison and treat those cases. Some form of restrictions and instructions for safe use and custody is imperative to reduce the burden of pesticide-related morbidity and mortality in the region, and restriction on availability of lethal pesticides is now considered as one of the basic approaches in suicide prevention.[11–13]

### Psycho-social stressors and gender specificity

Reduction of psycho-social stressors constitutes an important agenda in suicide-prevention strategy in developing countries.[14] The number of females who attempt DSH exceeds that of males who attempt DSH. It is revealed in the FGDs with the *Panchayat*s that different gender-specific causes were instrumental behind the female DSH attempts, like dowry demand and torture, mental and physical humiliation by the in-laws, derogatory behaviors by alcoholic husband, or emotional or economic distress resulting from extramarital relations of the husband. In the males, on the other hand, unsuccessful examination or love affairs and economic hardship were more common. It was also stated that in most of the females, there was no intention to die; rather, they used pesticides in a manipulative way to enforce threat on the family members to overcome distressing situations that they could not negotiate in a normal way. Examination and interview of many DSH cases in the field revealed this truth also. In this sense, DSH in some acted as a cultural mode of communication that follows the mechanism of hysterical symptom formation. Economic standard of living is very poor in the region, and most of the families have economic distress in some form or the other. Dowry system is widely prevalent, and dowry demand was one of the most important causes of familial conflict. An interesting comment came from an FGD with a *Panchayat* member of Namkhana block, who admitted that self-poisoning by pesticide has become a fashion in the entire Sundarban region, and this popularity of pesticides is replacing the traditional methods (like hanging or burning) of self-harm in recent years. According to him, whenever people are subject to some stressful life situations, they ingest pesticide either to die or for a change (in the familial relationship dynamics) profitable to them. Meetings with pesticide shop owners revealed that quite often, females or children went to the shops to buy pesticides as instructed by their parents. This type of practice also provides ready accessibility to lethal poisons even when they are not available in the home.

### Lack of education on safe use of pesticides

Information regarding potential health hazards of pesticides is nonexistent in the entire area. Most of the farmers never use musk, gloves and eye-protectors while spraying pesticides in the field. Nonfunctioning and complete inattention of the local agricultural office in this regard is a serious drawback. It is felt that the easy availability of these compounds acts as a catalyst to facilitate self-harm among the vulnerable subjects. Easy access to pesticide poisons is also linked with DSH motives and high impulsivity.[5]

Agriculture is the most common occupation in our country, and agrochemical pesticides were associated with high morbidity and mortality in the DSH cases, particularly from rural areas.[9] Therefore, an urgent intervention program is needed to reduce this preventable morbidity and mortality. An inter-sectorial program involving local agricultural office (for farmer's education on safe use and custody and health hazards of pesticides) and block primary health center (community awareness and psycho-social intervention) is the ideal goal for a community mental-health program in this rural region. Restricted use and sale of pesticides,[15,16] improved awareness regarding safe custody of pesticides, using less harmful compounds for agricultural purposes, rules for banning unlicensed shops that sell pesticides illegally may be effective and should be encouraged. Public health advocacy to prevent poisoning by indigenous substances like yellow oleander seed is also warranted. At the same time, improved hospital infrastructure for proper medical care of the poisoning cases[17] and timely psycho-social intervention at the community level will be beneficial.

The study has a number of limitations. Canning II, Basanti and Gosaba show very poor data-keeping, mainly because of improper hospital infrastructure in those remote rural areas. Moreover, for various reasons, not all cases of DSH attended hospitals; therefore, the actual number is well in excess of what is recorded in this study.

## References

[1] Phillips M (2004). Suicide prevention in developing countries: Where should we start?. World Psychiatry.

[2] Vander Hoek W, Konradsen F, Athukorala K, Wanigadewa T (1998). Pesticide poisoning:a major health problem in Sri Lanka. Soc Sci Med.

[3] Gunnell D, Eddleston M (2003). Suicide by intentional ingestion of pesticides: A continuing tragedy in developing countries. Int J Epidemiol.

[4] Mohanty MK, Kumar V, Bastia BK, Arun M (2004). An analysis of poisoning deaths in Manipal, India. Vet Hum Toxicol.

[5] Joseph A, Abraham S, Muliyil JP, George K, Prasad J, Minz S (2003). Evaluation of suicide rates using verbal autopsies, 1994-9. BMJ.

[6] Aaron R, Joseph A, Abraham S, Muliyil J, George K, Prasad J (2004). Suicides in young people in rural southern India. Lancet.

[7] Srinivas Rao Ch, Venkateswarlu V, Surender T, Eddleston M, Buckley NA (2005). Pesticide poisoning in South India: Opportunities for prevention and improved medical management. Trop Med Int Health.

[8] Chowdhury AN, Chowdhury S, Chakraborty A (1999). Eco-stress, quality of life and mental health in Sundarban delta of India. Int Med J.

[9] Eddleston M (2000). Patterns and problems of deliberate self-poisoning in developing world. QJM.

[10] Chowdhury AN, Sanyal D, Dutta SK, Weiss MG (2003). Deliberate self-harm by ingestion of poisons on Sagar Island in Sundarban delta, India. Int Med J.

[11] Lin JJ, Lu, TH (2006). Association between the accessibility to lethal methods and method-specific suicide rates: An ecological study in Taiwan. J Clin Psychiatry.

[12] Ohberg A, Lonnqvist J, Sama S, Vuori E, Penttila A (1995). Trends and availability of suicide methods in Finland: Proposals for restrictive measures. Br J Psychiatry.

[13] Bowles J, Diekstra R, Gulbinat R, Leo D, Kienhorst I Suicide in Western Samoa:an example of a suicide prevention programme in a developing country. Preventive Strategies on Suicide.

[14] Konradsen F, Hoek W, Peiris P (2006). Reaching for the bottle of pesticide-a cry for help: Self-inflicting poisoning in Sri Lanka. Soc Sci Med.

[15] Mann JJ, Apter A, Bertolote J, Beautrais A, Currier D, Haas A (2005). Suicide prevention strategies: A systematic review. JAMA.

[16] Nandi DN, Mukherjee SP, Banerjee G, Boral GC, Chowdhury AN (1979). Is suicide preventable by restricting the availability of lethal agents? A survey of West Bengal. Indian J Psychiatry.

[17] Rihmer Z (1996). Strategies of suicide prevention: Focus on health care. J Affect Dis.

